# Evolution of Hip Arthroscopy in Modern Orthopedic Practice

**DOI:** 10.7759/cureus.111657

**Published:** 2026-06-28

**Authors:** Muhammad Yamaan Khan, Raaed Khan, Hani Shaikh, Nadiya A Persaud, Sapna Rama

**Affiliations:** 1 Research, Orlando College of Osteopathic Medicine, Winter Garden, USA; 2 Research, University of California San Diego, San Diego, USA; 3 College of Public Health, University of South Florida, Tampa, USA

**Keywords:** femoroacetabular impingement, hip arthroscopy, joint preservation, labral repair, minimally invasive surgery

## Abstract

Hip arthroscopy has evolved from early endoscopic concepts into a modern minimally invasive orthopedic surgical technique. This review examines the historical progression of hip arthroscopy, beginning with foundational developments in endoscopic visualization in the 19th and early 20th centuries and advancing through key milestones that enabled safe access to the hip joint. Early limitations related to joint anatomy and visualization were gradually overcome through innovations in traction techniques, portal placement, and instrumentation, facilitating the transition from a primarily diagnostic modality to a reliable therapeutic approach. The late 20th century marked a period of rapid advancement, with increasing recognition of intra-articular pathology such as acetabular labral tears and femoroacetabular impingement (FAI). These developments contributed to the expansion of hip arthroscopy as a commonly utilized method for addressing a wide range of intra-articular and extra-articular conditions. In the modern era, technological innovations, including high-definition imaging, miniaturized instrumentation, and needle-based arthroscopy systems, have enhanced surgeons' ability to diagnose and treat hip pathology while potentially improving procedural efficiency and safety. Emerging clinical evidence and contemporary surgical experience suggest favorable patient outcomes associated with these advances, although outcomes vary depending on patient selection, pathology, and surgical technique. Additionally, evolving surgical philosophies have emphasized joint preservation, with a shift toward labral repair and reconstruction, capsular management, and comprehensive treatment of underlying pathology. As indications continue to expand and techniques become increasingly refined, hip arthroscopy remains a dynamic and rapidly advancing field with significant implications for patient care and future orthopedic practice.

## Introduction and background

Hip arthroscopy is a minimally invasive surgical technique used for the diagnosis and treatment of a wide range of intra- and extra-articular hip pathologies. As the understanding of hip pathology has advanced and demand for joint-preserving treatment options has increased, hip arthroscopy has become an important component of modern orthopedic practice. This approach provides access to key structures of the femoroacetabular joint, including the acetabular labrum, articular cartilage, joint capsule, and peritrochanteric space. In contemporary practice, hip arthroscopy is commonly utilized to manage conditions such as femoroacetabular impingement (FAI), labral tears, chondral injury, microfracture defects, greater trochanteric pain syndrome, snapping hip syndrome, and piriformis syndrome [1].

Modern arthroscopy employs an arthroscope equipped with a camera and light source, introduced through small incisions typically placed in the anterolateral and posterolateral regions surrounding the greater trochanter. Most procedures involve two to five small portal incisions, approximately 5-7 mm in diameter, which allow for visualization of the joint and the introduction of specialized instruments such as motorized shavers, burrs, suture passers, anchors, and probes [2]. Unlike more superficially accessible joints, the hip is a deep-seated and highly constrained ball-and-socket joint, making visualization and instrument access technically challenging and historically limiting the development of arthroscopic techniques. Joint distraction through traction is routinely used to increase the intra-articular space, facilitating improved visualization and safe instrumentation [3,4].

As a minimally invasive approach, hip arthroscopy is associated with reduced surgical morbidity, including lower risk of infection and blood loss, as well as decreased postoperative pain, scarring, and fibrosis. Enhanced visualization provided by camera-guided techniques improves surgical precision and accuracy. Additional benefits include shorter hospital stays and faster recovery times compared to traditional open procedures [5,6].

The utilization of hip arthroscopy has increased substantially over the past two decades, driven by advancements in imaging, instrumentation, and surgical technique, as well as improved understanding of hip pathology. In the United States, analyses of national insurance and administrative database records have demonstrated marked growth in procedural volume, with reported increases of 365% between 2004 and 2009 and an additional 200% increase from 2010 to 2014 [7]. Clinical outcomes are generally favorable, particularly in the treatment of FAI and labral pathology, with reported success rates of 85-90% and return-to-sport rates ranging from 70-93% among athletes [8,9].

Despite these benefits, complications can occur and include transient neuropraxia, nerve injury, fluid extravasation, infection, and thromboembolic events [10]. Postoperative recovery typically involves a period of protected weight bearing for 2-6 weeks, followed by structured physical therapy, with return to athletic activity generally expected within 4-6 months [11].

Methodology

This review provides a comprehensive overview of the literature regarding the historical evolution, technological advancements, and contemporary applications of hip arthroscopy. Relevant literature was identified through searches of electronic databases, including PubMed and Google Scholar, using keywords such as “hip arthroscopy”, “history of arthroscopy”, “femoroacetabular impingement”, “labral reconstruction”, “capsular management”, and “minimally invasive orthopedic surgery”. Additional articles were identified through manual review of the reference lists of relevant publications.

Articles were selected based on their relevance to the historical development, technological innovations, surgical techniques, biomechanical concepts, and clinical applications of hip arthroscopy. Priority was given to peer-reviewed journal articles, foundational studies, review articles, and recent literature describing contemporary advancements in the field. English-language publications deemed relevant to the objectives of this review were considered for inclusion.

Given the narrative nature of this review, a formal systematic search strategy, predefined inclusion and exclusion criteria, study screening process, Preferred Reporting Items for Systematic Reviews and Meta-Analyses (PRISMA) methodology, and risk-of-bias assessment were not performed. As such, article selection was based on relevance to the topics discussed rather than a systematic evaluation of all available literature. This review is subject to limitations, including potential selection bias and the absence of a formal systematic search, study screening process, and risk-of-bias assessment.

## Review

Early foundations

**Figure 1:**
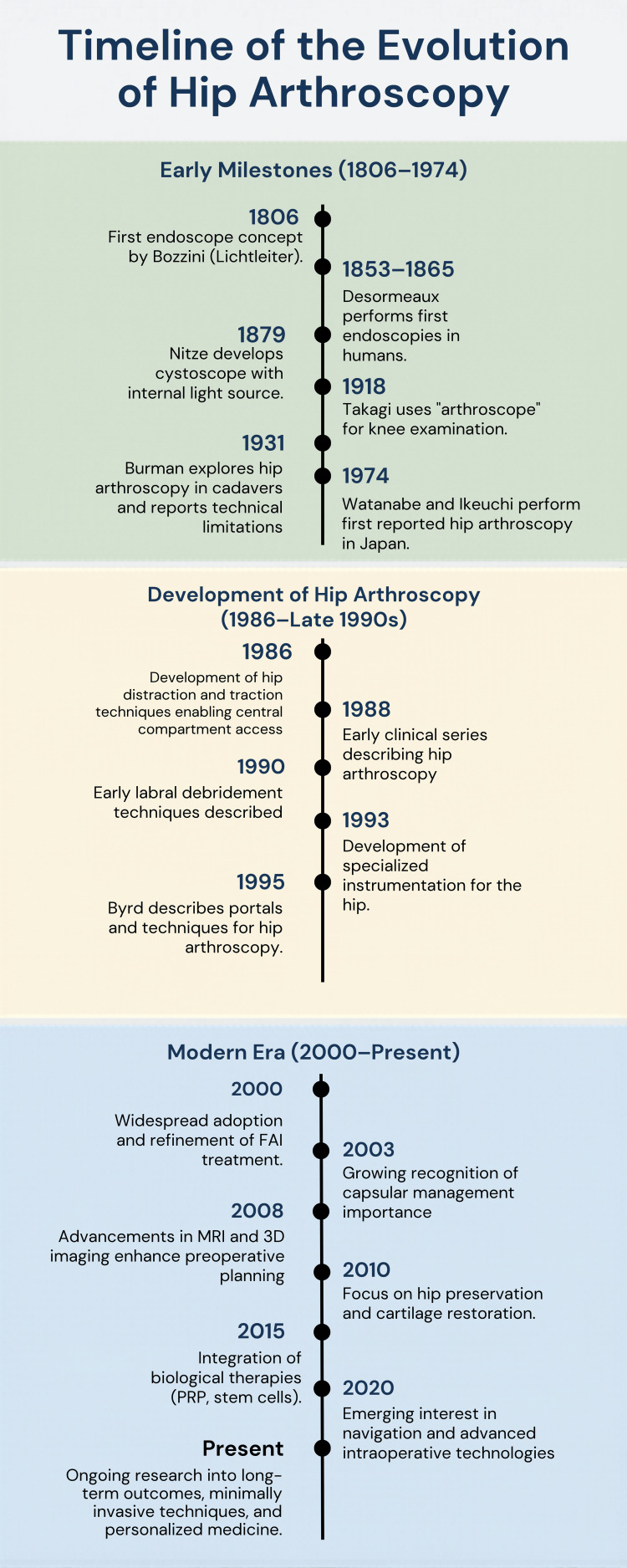
Timeline of the evolution of hip arthroscopy FAI: femoroacetabular impingement; MRI: magnetic resonance imaging; PRP: platelet-rich plasma. Figure created by Nadiya A. Persaud using Canva Pro (Canva Pty Ltd., Sydney, Australia), based on information synthesized from [2,12-30].

To contextualize the role of arthroscopic surgery in the modern era, it is important to examine the historical development of minimally invasive, endoscopic, and arthroscopic techniques (Figure 1).

The development of the Lichtleiter by Philipp Bozzini marked one of the earliest advances in endoscopy, enabling illumination and visualization of internal anatomical structures [12]. This innovation laid the foundation for endoscopic procedures, which were subsequently applied in urologic and gynecologic practice over the following century [12].

Advancements in minimally invasive techniques continued into the early 20th century. In 1901, Hans Christian Jacobeus reported a series of cases utilizing minimally invasive approaches for abdominal and thoracic examinations in humans [13]. This work was followed by the first laparoscopy performed in the United States by Bertram Bernheim at Johns Hopkins in 1911 [13].

These developments facilitated the application of endoscopic techniques to joint evaluation. In 1912, Severin Nordentoft proposed the use of an endoscopic method, termed arthroscopia genu, for the visualization of meniscal pathology, marking one of the earliest descriptions of arthroscopy [14]. Building upon this concept, Kenji Takagi utilized a cystoscope to examine the knee joint in cadaveric specimens and later developed a refined arthroscope with a smaller diameter, enabling improved access to the joint cavity. Takagi applied this technique clinically to identify intra-articular pathology, including tuberculous arthritis, which was prevalent at the time [15].

Shortly thereafter, Eugene Bircher published early clinical applications of knee arthroscopy in 1921 and 1922, further advancing the field [16]. Takagi continued to refine arthroscopic techniques and, in 1933, presented his findings at the Japanese Orthopaedic Association. Takagi’s subsequent work helped establish arthroscopy as a clinical tool for evaluating and treating various pathologies, including Charcot joints, tuberculous arthritis, and infectious arthritis [15].

The dissemination of arthroscopic techniques accelerated in the mid-20th century. In 1963, Robert Jackson traveled to Tokyo to study under Masaki Watanabe, a former student of Takagi. Upon returning, Jackson introduced arthroscopy to North America, contributing to its broader adoption [14]. This momentum culminated in the establishment of the International Arthroscopy Association in 1974, which facilitated global collaboration and expanded the application of arthroscopy to additional joints, including the hip [17].

Although arthroscopy gained acceptance relatively quickly in the knee, application to the hip joint progressed more slowly. Unlike the knee, the hip is a deeply situated, highly congruent ball-and-socket joint surrounded by substantial soft tissue and a thick capsule, making visualization and instrument access considerably more challenging. Furthermore, the narrow intra-articular space often requires joint distraction to create sufficient working room for arthroscopic instrumentation. Early arthroscopic equipment lacked the specialized instrumentation and visualization capabilities necessary to safely navigate the complex anatomy of the hip, further limiting its clinical application. As a result, the development and widespread adoption of hip arthroscopy lagged behind that of knee arthroscopy until advances in traction techniques, portal placement, and instrument design enabled safe and reproducible access to the joint [18].

Innovations in 1980s-1990s

By the late 1990s, acetabular labral tears were widely recognized as a significant source of hip pain and a contributor to degenerative intra-articular changes. This evolving understanding shifted clinical focus toward intra-articular hip pathology and established an early foundation for the expansion of hip arthroscopy as both a diagnostic and therapeutic modality, particularly in the management of labral tears and related conditions [19].

Earlier advancements further supported this transition. In 1986, Suzuki demonstrated the reliability of hip arthroscopy in identifying acetabular labral tears, offering a diagnostic advantage over arthrography [16]. This approach enabled more accurate visualization of intra-articular pathology and facilitated the identification of lesions that were previously difficult to detect, thereby reinforcing the clinical value of arthroscopy in the evaluation of hip disorders [16].

In parallel, advancements in traction techniques improved the accuracy and feasibility of hip arthroscopy. The development of controlled distraction methods enabled surgeons to achieve hip joint separation of approximately 8-10 mm, facilitating reliable access to the central compartment [2]. This represented a significant technical milestone, as limited joint space had previously restricted safe visualization and therapeutic intervention. As a result, the potential applications of hip arthroscopy expanded considerably [20].

In 1987, Glick et al. described reproducible techniques for establishing lateral portals in hip arthroscopy [21]. Their work outlined patient positioning and traction methods that facilitated improved access to the central compartment, building upon prior technical advancements. These developments enabled more consistent and safer entry into deeper regions of the hip joint, representing one of the earliest standardized approaches to hip arthroscopy comparable to modern techniques. This standardization contributed to the broader adoption of hip arthroscopy within the orthopedic field [21].

The 1990s were marked by continued expansion in hip arthroscopy, driven by the development of specialized instrumentation, refinement of traction techniques, increased research activity, and growing recognition of its therapeutic applications. During this period, professional organizations such as the International Society for Arthroscopy, Knee Surgery, and Orthopaedic Sports Medicine were established (1995), further promoting education, collaboration, and the advancement of arthroscopic techniques within the orthopedic community [17].

Ultimately, the collective impact of these scientific and organizational advancements established hip arthroscopy as an evidence-based surgical discipline, laying the foundation for its rapid expansion in the early 2000s.

Modern advancements (2000-2026)

Hip arthroscopy has evolved from a primarily diagnostic modality into a comprehensive, minimally invasive technique capable of addressing both intra- and extra-articular pathologies. In contemporary practice, it is routinely utilized to evaluate unexplained hip or groin pain, as well as to assess conditions such as joint effusions and synovitis [19]. A key advantage of arthroscopy is its ability to provide direct visualization of intra-articular structures, particularly in cases where conventional imaging is inconclusive.

Recent technological advancements have further expanded the clinical utility of hip arthroscopy, including the development of minimally invasive systems such as the NanoScope Operative Arthroscopy System (Arthrex, Inc., Naples, USA). This innovation incorporates a miniaturized, chip-on-tip camera design, typically measuring approximately 2 mm in diameter, which allows for smaller portal creation and reduced soft tissue disruption compared to traditional 4-5 mm rod-lens arthroscopes [24]. Early clinical experience and emerging evidence suggest that these systems may improve procedural efficiency and facilitate in-office or minimally invasive diagnostic evaluation while potentially reducing healthcare costs in selected settings. However, long-term comparative outcome data remain limited. Additionally, the reduced instrument size may facilitate portal placement and decrease the risk of soft tissue trauma and iatrogenic cartilage injury while maintaining visualization of intra-articular structures and periarticular soft tissues [21-23].

Additionally, diagnostic needle arthroscopy has demonstrated accuracy comparable to standard surgical arthroscopy and, in select cases, superior performance relative to magnetic resonance imaging for evaluating intra-articular pathology, while also offering a potentially cost-effective alternative. The continued miniaturization of arthroscopic technology has facilitated a transition from traditional operating room-based procedures to in-office diagnostic settings. This shift has been associated with reduced procedural morbidity, faster recovery times, improved efficiency, and decreased resource utilization, ultimately enhancing accessibility and enabling more timely evaluation and management of patients [24,25]. In-office diagnostic arthroscopy has been shown to be a safe and effective modality, with multicenter prospective data supporting its diagnostic accuracy relative to both surgical arthroscopy and magnetic resonance imaging [26]. However, several limitations remain. Successful implementation requires familiarity with specialized equipment and procedural techniques, which may present a learning curve for practitioners. Patient tolerance may vary depending on the joint being evaluated and the complexity of the examination, and needle arthroscopy remains primarily a diagnostic tool with limited therapeutic capability compared to conventional operative arthroscopy. As a result, patients requiring definitive intervention may still require subsequent surgical arthroscopy [24-26].

Alongside technological advancements, the surgical philosophy of hip arthroscopy has evolved toward joint preservation rather than excision. FAI has emerged as a primary indication for hip arthroscopy, reflecting a transition from traditional open surgical dislocation techniques to minimally invasive arthroscopic management, including cam and pincer lesion correction. Arthroscopy is now widely utilized for the treatment of FAI and associated pathology, providing direct visualization of both intra- and extra-articular structures while supporting modern joint-preserving approaches [2,28].

Hip arthroscopy is now well established as a joint-preserving procedure, with arthroscopic osteoplasty demonstrating outcomes comparable to open surgical techniques. Early approaches involving labral excision for anterior, non-traumatic tears were associated with suboptimal outcomes, leading to a paradigm shift toward labral preservation and repair [29,30]. Current evidence emphasizes that addressing the underlying impingement pathology is critical for optimizing clinical outcomes and reducing the risk of recurrence [30].

As surgical techniques have advanced, labral management has evolved in a stepwise manner from debridement to repair and, more recently, to reconstruction. Initial reports of arthroscopic labral reconstruction emerged in the early 2010s, with broader adoption occurring for the management of irreparable or nonfunctional labral tears [31,32]. Contemporary hip preservation strategies emphasize restoration of labral function whenever possible. Labral repair remains the preferred treatment for viable, repairable labral tissue in most primary procedures, as preservation of the native labrum helps maintain the suction seal and biomechanical function of the hip joint [33]. In contrast, labral reconstruction is generally indicated when the labrum is irreparable due to severe degeneration, calcification, hypoplasia, deficiency, or prior failed treatment and has demonstrated favorable clinical outcomes in both primary and revision settings [34,35]. High-volume hip arthroscopists increasingly favor reconstruction over debridement in cases where labral preservation is not feasible, as clinical and biomechanical studies have shown improved restoration of hip biomechanics and patient-reported outcomes. Additionally, advancements in technique, such as knotless pull-through fixation, have further refined labral restoration and enhanced surgical reproducibility [31].

Hip joint stability is determined not only by osseous anatomy but also by key soft-tissue structures, including the acetabular labrum, joint capsule, ligamentum teres, and surrounding musculature such as the iliopsoas. These structures function synergistically to maintain the labral suction seal, restrict excessive motion, and provide both static and dynamic stabilization of the hip [32]. This improved understanding of hip biomechanics has led to a shift in modern surgical practice away from routine capsulotomy without repair toward techniques that preserve capsular integrity. Capsular repair, plication, and reconstruction, often performed in conjunction with femoroplasty, have been increasingly adopted to minimize the risk of iatrogenic instability and optimize patient outcomes [33,34]. Particular attention to capsular management is warranted in patients at increased risk for postoperative instability, including those with generalized ligamentous laxity and borderline acetabular dysplasia, as preservation or restoration of soft-tissue constraints may be especially important for maintaining hip stability in these populations [35-38].

Advances in surgical technique and perioperative technology have significantly improved the safety of hip arthroscopy. Refinements in traction protocols, patient positioning, fluid management systems, and the sequencing of extra-capsular procedures have contributed to a reduction in complications such as traction-related neuropraxia, fluid extravasation, and articular surface injury [39,40]. As surgeons' experience has increased, the scope of arthroscopic intervention has expanded to include more complex reconstructive procedures. This progression has been accompanied by a decrease in fundamental technical complications and a shift toward addressing the challenges associated with advanced surgical techniques [41].

The indications for hip arthroscopy have expanded to include complex reconstructive procedures, with applications extending to periarticular tendon repair, fracture reduction and fixation, management of femoral head osteochondritis dissecans, and endoscopic treatment of recalcitrant osteitis pubis. These developments highlight the increasing versatility of hip arthroscopy in contemporary orthopedic practice [31].

Future directions

The future of hip arthroscopy is expected to be driven by continued technological innovation, refinement of surgical techniques, and an improved understanding of hip biomechanics. Advancements in imaging and intraoperative guidance, along with the development of minimally invasive and needle-based arthroscopic systems, are likely to expand the role of in-office procedures and improve diagnostic accuracy while reducing healthcare costs [25-27]. Additionally, emerging strategies in labral reconstruction, capsular management, and biologic augmentation, including platelet-rich plasma and stem cell therapies, are being investigated for their potential to enhance tissue healing and improve clinical outcomes. However, the current evidence supporting biologic augmentation remains heterogeneous, with variability in study design, biologic preparation methods, treatment protocols, and reported outcomes. Consequently, the role of platelet-rich plasma and stem cell therapies in hip arthroscopy remains investigational, and further high-quality studies are needed to establish their efficacy and optimal clinical applications [42-44]. As the scope of hip arthroscopy continues to broaden to include more complex reconstructive procedures, further high-quality studies will be essential to establish standardized protocols, optimize patient selection, and evaluate long-term efficacy and safety.

## Conclusions

Hip arthroscopy has evolved from an experimental concept into a widely utilized minimally invasive technique for the diagnosis and treatment of diverse hip pathologies. Advances in technology, surgical instrumentation, and understanding of hip biomechanics have expanded its role in joint preservation, particularly through labral repair and reconstruction, capsular management, and correction of FAI. Despite these advances, challenges remain, including the technical learning curve associated with increasingly complex procedures, the management of revision cases, and the importance of appropriate patient selection to optimize outcomes. As indications continue to expand, particularly for complex reconstructive techniques and biologic augmentation strategies, long-term prospective studies will be essential to evaluate durability, safety, and clinical effectiveness. Continued research and innovation will play a critical role in refining surgical techniques, minimizing complications, and defining the future role of hip arthroscopy in orthopedic practice.
